# Clinical Preventive Services in Guatemala: A Cross-Sectional Survey of Internal Medicine Physicians

**DOI:** 10.1371/journal.pone.0048640

**Published:** 2012-10-31

**Authors:** Juan E. Corral, Lauren D. Arnold, Erwin E. Argueta, Akshay Ganju, Joaquín Barnoya

**Affiliations:** 1 Research Department, Cardiovascular Unit of Guatemala, Guatemala City, Guatemala; 2 Division of Public Health Sciences, Department of Surgery, Washington University in St. Louis, School of Medicine, St. Louis, Missouri, United States of America; University of Washington, United States of America

## Abstract

**Background:**

Guatemala is currently undergoing an epidemiologic transition. Preventive services are key to reducing the burden of non-communicable diseases, and smoking counseling and cessation are among the most cost-effective and wide-reaching strategies. Internal medicine physicians are fundamental to providing such services, and their knowledge is a cornerstone of non-communicable disease control.

**Methods:**

A national cross-sectional survey was conducted in 2011 to evaluate knowledge of clinical preventive services for non-communicable diseases. Interns, residents, and attending physicians of the internal medicine departments of all teaching hospitals in Guatemala completed a self-administered questionnaire. Participants’ responses were contrasted with the Guatemalan Ministry of Health (MoH) prevention guidelines and the US Preventive Services Task Force (USPSTF) recommendations. Analysis compared knowledge of recommendations within and between hospitals.

**Results:**

In response to simulated patient scenarios, all services were recommended by more than half of physicians regardless of MoH or USPSTF recommendations. Prioritization was adequate according to the MoH guidelines but not including other potentially effective services (e.g. colorectal cancer and lipid disorder screenings). With the exception of colorectal and prostate cancer screening, less frequently recommended by interns, there was no difference in recommendation rates by level.

**Conclusion:**

Guatemalan internal medicine physicians’ knowledge on preventive services recommendations for non-communicable diseases is limited, and prioritization did not reflect cost-effectiveness. Based on these data we recommend that preventive medicine training be strengthened and development of evidence-based guidelines for low-middle income countries be a priority.

## Introduction

Guatemala, a low/middle income country (LMIC), has moved from the first stage of the epidemiologic transition, where infectious diseases and nutritional deficiencies prevail, to the second stage, where non-communicable diseases (NCD) are on the rise.[1]–[3] Today, NCD are the leading cause of death in Guatemala, accounting for nearly half of the country’s total deaths.[4]–[6] Cardiovascular diseases (CVD) and cancer account for half of Guatemala’s NCD deaths.[4]–[6].

High-income countries have made some progress in controlling NCD. [7], [8]_ENREF_9 Risk factor modification and other preventive strategies, such as screening, have made significant contributions to reducing morbidity and mortality when compared to advances in medical and surgical interventions.[9]–[13]_ENREF_13 Regardless of a country’s developmental status, the World Health Organization advocates prevention as the most cost-effective way of controlling chronic diseases, such as cancer and CVD. [14] Therefore, preventive services have become an important aspect of clinical and public health practice. [15] Clinical preventive services that individualize interventions based on risk assessment can substantially reduce costs (e.g. time, money, personnel) compared to a mass prevention approach. [16] This may be particularly important in resource-limited countries, such as Guatemala.

In February 2011, the Guatemalan Ministry of Health (MoH) released the Guidelines for Prevention, Detection, Evaluation, and Treatment of NCD. [17] These were developed based on reports from different associations (e.g. The U.S. Joint National Committee on Prevention, Detection, Evaluation, and Treatment of High Blood Pressure) and other countries (U.S., Mexico, Costa Rica and Colombia) rather than a local evidence-based analysis. Details on the selection process used to determine which services should be offered, starting age, and frequency are missing. Furthermore, these guidelines were designed for rural care at health posts and centers, where nurses are the main providers of care under limited resources. [17], [18].

The United States Preventive Services Task Force (USPSTF) systematically reviews and publishes evidence-based recommendations and classifies strategies according to strength of the available evidence: “A” (strongly recommends), “B” (recommends), “C” (recommends against routine use but can be provided on an individual basis), “D” (recommends against), or “I” (insufficient evidence to recommend for or against). [19] These recommendations, although designed for application in the U.S., represent a rigorous appraisal of peer-reviewed evidence and may serve for other healthcare systems to evaluate services’ cost-effectiveness.

Among clinical preventive services, screening and brief intervention for tobacco use are the single most cost-effective methods to prevent NCD. [20], [21] Clinicians should ask all patients about tobacco use, provide counseling, and offer cessation pharmacotherapy. Unfortunately, worldwide, cost-effective services like tobacco screening and counseling are not frequently provided. [20], [22] Furthermore, less cost-effective services and even those that should not be recommended are offered routinely without net benefit to the patient and leading to unnecessary testing. [23].

In Guatemala less than 5% of the population receives outpatient care from a physician. [4] Internists, as the most trained personnel providing primary care in the country, should become advocates to ensure availability and affordability of preventive services. Furthermore, seven out of eleven members of the expert panel writing the Guidelines for Prevention, Detection, Evaluation, and Treatment of NCD were internists. [17].

To explore knowledge about preventive services among Guatemalan internal medicine physicians, a self-administered survey was given to interns, residents, and attendings in all teaching hospitals nationwide. It was expected that physicians should be knowledgeable of the MoH screening recommendations. Additionally, given the epidemiologic transition, other services like colorectal cancer and lipid disorder screenings (not included in the 2011 MoH guidelines) have potential benefit in Guatemala. Therefore we aimed to compare physicians’ answers to the MoH guidelines but also included other services reviewed in the USPSTF recommendations.

## Methods

All ten hospitals that provide internal medicine training in Guatemala were invited to participate in the study: six in Guatemala City, the other four in Antigua Guatemala, Cuilapa, Quetzaltenango, and Escuintla. The sample included “public” hospitals (operated by the MoH, that provide all services for free, with no insurance required) and “other” hospitals (the Guatemalan Social Security Institute (IGSS), the Military Hospital, and two private hospitals with residency programs, all of them located in Guatemala City). House staff were classified as interns (last-year medical students), residents, and attending physicians. The latter included department directors and program directors, as they are required to provide patient care and have teaching responsibilities regardless of their administrative roles.

Four trained surveyors approached physicians at classrooms after ground rounds. Those that did not attend rounds were approached at their regular workplaces. Surveyors explained the purpose of the study, asked willingness to participate, and presented the questionnaire. The questionnaire was anonymous and self-administered. Prior to implementation, it was pilot tested in a group of medical students and physicians unrelated to any of the training programs included in our protocol.

The questionnaire included: demographics, time committed to outpatient care, knowledge of leading causes of death in Guatemala, guidelines followed when recommending preventive services (e.g. MoH, USPSTF), and questions on selected NCD preventive services. Questions about preventive services included those options available in Guatemala to prevent the most relevant NCD (CVD, cancer, diabetes).

Service questions began with a brief generic scenario. Each scenario (10 in total) included an asymptomatic adult (age >18 years), with no relevant family history, followed by a question on whether or not the service would be recommended. Those who answered affirmatively were then asked about starting age and frequency (open-ended questions), service of choice (e.g. mammogram or clinical breast exam), and perceived availability (e.g. “Chances of receiving diet counseling at your institution are: very likely, likely, neither likely nor unlikely, unlikely, almost impossible”). All questions were written in conditional tense (“should be done”) without describing the environment (except for the availability in their corresponding hospital). Answers were compared with the MoH Guidelines and USPSTF Recommendations. Finally the survey also assessed perceived barriers to providing preventive services, opinions on who should develop guidelines, and who should train Guatemalan physicians on NCD prevention.

Based on pilot testing the instrument and from our previous experience, lack of agreement in guidelines used (e.g. MoH, USPSTF), and a large percentage of physicians not using any guidelines were expected. Initially, participants’ answers were evaluated using the percentage of staff that recommended all services suggested in the Guatemalan guidelines assuming that appropriately trained physicians would prioritize all services recommended by the MoH. Secondary analysis included two additional preventive services (colorectal cancer and lipid disorders screening) due to their proven cost-effectiveness in other populations and potential benefit in Guatemala, despite not being mentioned in the MoH guidelines. [4], [5] There is still an ongoing debate if colorectal cancer screening should be promoted in low-resource countries. [24].

To ensure data quality, a double entry system and random checks were performed. Descriptive statistics were generated, with Mann-Whitney, Chi-square, or Fisheŕs exact tests to determine significant differences. Resident and intern responses to time committed to outpatient care were merged since both only see patients at teaching hospitals while attendings also work at other private clinics or hospitals. Analyses were done using Stata/SE 11.2.

The study protocol was approved by the institutional review boards of Hospital Roosevelt, Hospital General San Juan de Dios, Hospital Regional de Occidente, Hospital Nacional Regional de Escuintla, Hospital Nacional Pedro de Bethancourt and the Washington University in St. Louis School of Medicine.

## Results

Nine of the ten invited hospitals participated in this study (one public hospital declined to participate without disclosing reasons). Participating hospitals included a total of 467 internal medicine physicians. After excluding those on vacation, sick leave, or suspension, 443 participants remained. Of these physicians, 394 (88.84%) participated. Response rate varied by level of training: 96.74% (n = 89) interns, 94.74% (198) residents, and 75.35% (107) attending physicians. Lack of time and interest were the most frequently cited reasons not to participate. Respondents’ characteristics are presented in Table 1. Attending physicians had been working, on average, 12.01±10.43 years at their hospital.

**Table 1:** Internal medicine physicians’ demographics.

n = 394	
**Age**, median (IQR)	28 (25–34)
**Gender**, n (%)	
Male	244 (61.93)
Female	150 (38.07)
**Training level**, n (%)	
Intern	89 (22.59)
Resident^a^	198 (50.25)
Attending^b^	107 (27.16)
**Completed medical school in Guatemala,** c n (%)	351 (89.08)
**Hospital**, n (%)	
Public	286 (72.59)
Other	108 (27.41)
**Percentage of working time devoted to outpatients**, median hours (IQR)	20 (0–50)

Guatemala. 2011.

a Eighty three (41.92%) first year residents, 57(28.79%) second year, 48(24.24%) third year, and 10(5.05%) chiefs of residents.

b Seventeen (15.89%) department directors, 63 (58.88%) program directors, and 27 (25.23%) floor attendings.

c Of those trained abroad, 14 (32.56%) trained in Cuba, 11 (25.58) in Honduras, 10 (23.25%) in El Salvador, and 8 (18.61) in other countries.

Time spent providing outpatient care differed by level of training and hospital type. Median (interquartile range, IQR) time spent by attendings (50%, 30–70%) was higher (p<0.001) compared to interns and residents (10%, 0–30%). Public hospital physicians (10%, 0–40%) spent less time with outpatients than those at other hospitals (30%, 10–60%, p<0.001). However, the percentage of asymptomatic patients seeking a health check was identical (p = 0.1) between attendings (10%, 0–20%) and interns and residents (10%, 0–20%). There was a significant difference in the amount of health check visits between physicians at public (10%, 0–10%) and at other hospitals (10%, 0–30%) (p<0.001). Twenty four participants (6.14%) estimated spending less than one minute discussing NCD screening per patient, 277 (70.84%) reported between one and five minutes, and 90 (23.02%) more than five minutes.

Regarding the leading causes of death, more than one-third (39.67%) of respondents underestimated the contribution of CVD and cancer, and overestimated infectious and perinatal contributors to the leading causes of deaths. Regarding preventive services, most respondents (247, 63.33%) answered that they were familiar with the American Heart Association, followed by the Guatemalan MoH (134, 34.36%), the American Cancer Society (69, 17.69%) and the USPSTF (25, 6.41%) recommendations. Forty four physicians (11.28%) did not knew any guidelines at all.

In response to simulated patient scenarios, all services were recommended by more than half of surveyed physicians, regardless of MoH recommendations or USPSTF grade (Table 2). The most frequently recommended services were tobacco cessation interventions and hypertension screening. Colorectal cancer screening had the lowest recommendation rates (Table 2). Services recommended by the Guatemalan MoH were recommended more frequently than other services (p<0.001, Table 3). However, when colorectal cancer screening and lipid disorder screening were considered recommended preventive services (both USPSTF Grade A) no difference was found. (p = 0.49, Table 3).

**Table 2:** Physicians’ recommendations for simulated cases and preventive service of choice.^a^

PreventiveService	Recommended byGuatemalan MoH	USPSTFGrade	Recommended bystaff, n = 394 (%)^b^	Service ofchoice	%	Availability at participating Hospitals, n = 9 (%)^c^
Ask about tobacco use	Yes	A	382 (97.45)	NA		9 (100)
Tobacco cessation intervention	Yes	A	372 (99.20)	Counseling only	59.36	9 (100)
				Counseling with pharmacotherary	18.32	0 (0)
				Nicotine replacement	12.03	0 (0)
				Varenicline	6.15	0 (0)
				Bupropion	2.14	0 (0)
				Other/NR	2.01	
Hypertension screening	Yes	A	385 (98.97)	NA		9 (100)
Cervical cancer screening	Yes	A	378 (95.94)	Pap-smear	92.59	9 (100)
				VIA^d^	6.61	7 (77.78)
				Other/NR	0.79	
Colorectal cancer screening	Not mentioned	A	218 (55.75)	Colonoscopy	50.68	8 (88.89)
				FOBT	40.72	5 (55.56)
				Sigmoidoscopy	5.43	5 (55.56)
				Other/NR	3.17	
Lipid disorders screening	Not mentioned	A	314 (80.51)	NA		8 (88.89)
Breast cancer screening	Yes	B	366 (93.13)	Clinical examination^d^	55.59	9 (100)
				Mammogram	41.42	7 (77.78)
				Other/NR	3.00	
CHD screening^e^	Not mentioned	D	237 (60.31)	NA		9 (100)
Healthy diet counseling	Yes	I	373 (94.67)	NA		9 (100)
Diabetes screening	Not mentioned	I	335 (85.46)	Fasting glucose	70.03	9 (100)
				Glycosilated hemoglobin	18.99	8 (88.89)
				Glucose tolerance test	7.12	9 (100)
				Other/NR	3.86	
Prostate cancer screening	Not mentioned	I	316 (81.65)	Prostate specific antigen	53.87	6 (66.67)
				Digital rectal exam	40.87	9 (100)
				Other/NR	5.26	

a All simulated cases were asymptomatic patients without any risk factors.

b Percentages were calculated with the staff that answered affirmatively divided by the staff that answered each question.

c Services are considered available if equipment or trained personnel are available regardless of tests being approved for screening purposes or personnel having dedicated time for preventive interventions.

d Using these screening methods is considered a grade I recommendation.

e Electrocardiogram for CHD screening.

MoH: Ministry of Health, USPSTF: U. S. Preventive Services Task Force, NA: Not Available, NR: No Response, VIA: Visual Inspection with Acetic Acid, FOBT: Fecal Occult Blood Testing, CHD: Coronary Heart Disease.

**Table 3:** Physicians’ recommendations for simulated cases stratified by Guatemalan MoH recommendations.^a^

Preventive service	% of staff that recommended all	p
Recommended by Guatemalan MoH	88.1	<0.001
Not recommended by Guatemalan MoH	38.7	
Recommended by Guatemalan MoH, colorectal cancer screening and lipid disorders screening.	48.69	0.49
Other services^b^	51.43	

a All simulated cases were asymptomatic patients without any risk factors.

b Other services are coronary heart disease, diabetes and prostate cancer screening.

MoH: Ministry of Health.

Colorectal and prostate cancer screening was the only area where recommendations differed significantly by level of training, with less frequent recommendation by interns (Table 4). Although public hospital physicians recommended all services less frequently, this was only significant for lipid disorders, diabetes, and colorectal and prostate cancer screening (Table 4).

**Table 4:** Percentage of physicians that recommended each preventive service, according to training level and hospital type.

	Training level				Hospital		
Preventive Service	Intern n = 89^a^	Resident n = 198	Attending n = 107	p	Public^b^ n = 286	Other^c^ n = 108	p
Ask about tobacco use	98.86	96.46	98.11	0.5	97.18	98.15	0.7
Tobacco cessation intervention	97.70	99.46	100.00	0.2	99.26	99.04	0.9
Hypertension screening	98.88	98.97	99.06	0.9	98.59	100.00	0.6
Cervical cancer screening	95.51	94.95	98.13	0.4	95.45	97.22	0.6
Colorectal cancer screening	40.91	54.08	71.03	<0.001	50.70	69.16	0.001
Lipid disorders screening	74.16	79.49	87.74	0.05	77.19	89.52	0.006
Breast cancer screening	95.51	91.37	94.39	0.4	91.58	97.22	0.05
CHD screening^d^	60.67	58.38	63.55	0.7	58.25	65.74	0.2
Healthy diet counseling	92.13	95.45	95.33	0.5	94.06	96.30	0.4
Diabetes screening	78.65	86.80	88.68	0.1	83.16	91.59	0.03
Prostate cancer screening	72.73	80.93	90.48	0.006	77.86	91.59	0.002

a Percentages were calculated with the staff that answered affirmatively divided by the staff that answered each question.

b Public hospitals are operated by the Ministry of Public Health and Social Welfare (MSPAS), all services are free of cost, no insurance required.

c This includes the Guatemalan Social Security Institute (IGSS), the Military Hospital and two private hospitals.

d Electrocardiogram for CHD screening.

Knowledge on when to begin screening was discordant with both MoH and USPSTF guidelines evaluated. Physicians recommended hypertension and cervical cancer screening to begin at later ages. Conversely lipid disorders, breast and colorectal cancers screening were offered earlier on simulated cases than on USPSTF recommendations (Table 5) (MoH guidelines do not include a starting age). Overall, physicians recommended services more frequently than both reference guidelines. Less than one quarter knew the appropriate frequency to repeat Pap-smears, visual inspection with acetic acid, lipid disorder screening, mammograms, colonoscopies, and sigmoidoscopies (Table 5).

**Table 5:** Physicians’ suggested starting age and frequency for each preventive service compared to Guatemalan MoH guidelines and USPSTF recommendations.

	Starting age			Frequency		
Preventiveservice	Physicians’recommendations^a^,years (SD)	Guatemalan MoHguidelines, years	USPSTFrecommendations,years	Physicians’recommendations,preferredfrequency (%)	GuatemalanMoH guidelines(% correct)	USPSTFrecommendations(% correct)
Ask about tobacco use				Annual (96.77)	Annual	Annual
Hypertension screening	26.26 (8.92)	20	18	Annual (98.42)	Annual	Annual
Cervical cancer screening	25.92 (8.89)	25 or after onset of sexual activity	21 or 3y after onset of sexual activity			
Pap-smear				Annual (91.33)	Every 3y (1.73)	Every 3y (1.73)^b^
VIA				Annual (84.00)	Every 3y (4.00)	NA
Lipid disorders screening^c^	32.39 (8.40)	NA	35	Annual (81.11)	NA	Every 5y (1.63)^b^
Breast cancer screening	37.46 (7.45)	NA	50			
Mammogram				Annual (73.51)	NA	Every 2y (13.25)
Clinical examination				Annual (95.57)	Annual	NA
Colorectal cancer screening	43.62 (7.58)	NA	50			
Colonoscopy				Every 5y (25.89)	NA	Every 10y (12.50)
FOBT				Annual (68.18)	NA	Annual
Sigmoidoscopy				Annual (66.67)	NA	Every 3y (16.67)
CHD screening^d^				Annual (65.95)	NA	NA
Healthy diet counseling				Annual (94.58)	Annual	NA
Diabetes screening				Annual (85.07)	NA	NA
Prostate cancer screening	44.39 (6.12)	NA	NA			
Prostate specific antigen				Annual (67.82)	NA	NA
Digital exam				Annual (63.64)	NA	NA

a Recommendations were asked with simulated case scenarios of asymptomatic patients without any risk factors that came annually for a health check.

b The frequency of screening is still uncertain.

c Lipid disorder screening questions were specific for male patients.

d Electrocardiogram for CHD screening.

MoH: Ministry of Health, NA: Not available, VIA: Visual Inspection with Acetic Acid, FOBT: Fecal Occult Blood Testing, CHD: Coronary Heart Disease.

 Regarding service of choice for screening in different hospital types, no significant difference was found between colonoscopy or fecal occult blood test (p = 0.4). However, there was a significant difference for breast (p = 0.04) and prostate cancer (p<0.001) screenings. Internists at public hospitals preferred the clinical breast and digital rectal examinations over mammography and prostate specific antigen more frequently than those at other hospitals.

Service availability at hospitals was for the most limited. Hypertension screening was perceived as the most available service followed by asking about tobacco use (93.6% and 77.6%, respectively). Lipid disorder, breast and cervical cancers, and CHD screenings were perceived available by half of respondents. Providing tobacco cessation pharmacotherapy was perceived as the least available preventive service (4.83% physicians) followed by colorectal cancer screening (23.24% physicians).

The main perceived barriers reported by physicians to their providing preventive services were lack of time (46.38%), inadequate patient resources (31.34%), and lack of patient interest (23.45%). Other barriers included low physician confidence and forgetfulness (11.83%).

Implementing a national NCD prevention program was considered to be the responsibility of the MoH by most physicians (87.86%). Likewise, 42.29% considered it is the MoH’s responsibility to improve preventive medicine education, followed by medical schools (29.71%), and the Guatemalan College of Physicians and Surgeons (13.71%). Twelve percent believed the medical staff should be responsible for improving preventive services education (interns 10.84%, residents 14.29%, and attendings 8.69%, p = 0.38).

## Discussion

Internal medicine physicians have limited knowledge on preventive services recommendations. In a resource-limited setting like Guatemala physicians should recommended preventive services based on the evidence available, encouraging practices proven to benefit the patient and discouraging those proven to harm or where the evidence is inconclusive.

Among all preventive services, asking about tobacco use and discussing smoking cessation interventions are the most cost-effective service to prevent NCD. [20], [21] Even though physicians were knowledgeable about this service, providing cessation pharmacotherapy was perceived as the least available service. Despite the evidence, cessation pharmacotherapy is found in less than one quarter of Guatemalan pharmacies and when found, unaffordable compared to the minimum daily wage. [25] However, training physicians in providing cessation services should lead to an increase in cessation attempts and demand for pharmacotherapy.

Colorectal cancer screening had the lowest recommendation rates of all. This probably relates to low perceived availability (considered the second least available service), the actual low availability (despite having equipment and trained personnel, colonoscopy and sigmodioscopy are reserved for diagnostic purposes) and the absence of recommendations in Guatemalan guidelines. Additionally, studies from U.S., France, Greece, Mexico, and Brazil have documented that physicians score lower in colorectal cancer screening knowledge and provision compared with other recommendations.[26]–[30] Medical education and training should also strive to discourage use of ineffective recommendations that Guatemalan physicians favor, particularly CHD screening with an electrocardiogram. Given the limited healthcare resources in Guatemala and, as our results yield, physicians perceive economic factors as one of the barriers to provide services, discouraging the use of such ineffective practices should prove particularly beneficial as a cost saving strategy.

In addition to inappropriate selection of services, better training as to the age for starting and frequency of screening are needed. This is crucial given that most patients visit a physician near the age to start screening for colorectal and breast cancers (average age at public hospitals’ internal medicine clinics 48.31±20.27 years old). [31], [32] In Guatemala, like in the U.S. and Mexico, recommendation practices improved with advanced training levels.[29], [33]–[35] However, given the relevance of recommendations, residents should receive appropriate training on preventive medicine early in their careers.

The most frequently perceived barriers to providing preventive services in Guatemala were lack of time and inadequate resources. In Latin America, accessibility and availability of quality services are perceived as the main barriers for screening. [36] Once these improve, patient resources and time limitations become important barriers, as has been the case in the U.S. with low-income patients. This might reflect patients’ tendency to present in “acute” situations rather than on an appointment basis, limited resources for out-of-pocket expenses, lack of spare time, and/or low educational level. [37].

In Guatemala, perceived availability of preventive services is determined by hospital type. Hospital type also determined the choice of screening test for breast and prostate cancers. In public hospitals clinical screening was preferred over mammography or prostate antigen. As opposed to private hospitals where patients pay with insurance or out-of-pocket, public hospitals are constrained by low availability of drugs and diagnostics, overcrowding, and long waiting times. [18] Overall, these circumstances explain, in part, the lower recommendation rates seen in public hospitals.

Physicians’ training with outpatients has been associated with better recommendations and provision of preventive services. [38], [39] Our results show that attendings spend significantly more time with outpatients than interns and residents. From a training perspective, interns and residents should be devoting more time in the outpatient clinic as this is where they will most likely practice after completing their residency. This “inpatient-centered” training disconnected from communities’ healthcare needs is the case in most LMICs. [40] Therefore, any approach to improve delivery of preventive services should include more outpatient exposure during internal medicine residency.

Finally, our study identifies some limitations of MoH guidelines as means promoting provision of prevention services. Recommendations might better be tailored to available epidemiologic data and cost-effectiveness analyses for Guatemala (rather than on other countries’ recommendations), and should state a position for all available preventive services (even those proven infective or not cost-effective) considering that physicians recommended them on simulated-case scenarios.

Our study has strengths and limitations. To our knowledge this is the first study to assess physicians’ knowledge of preventive services for NCD in a LMIC. This is particularly relevant. Early in the epidemiologic transition when incidence is rising, cost-effective healthcare strategies to prevent the NCD epidemic may be most appropriate. The objective of this study was to measure physicians’ knowledge, not actual practice. To address the latter, a different study design is required (e.g. chart review, patient surveys). Addittionally, our study based on the Guatemalan MoH guidelines and the USPSTF recommendations showed a substantial gap. A cascade model that uses, “do what you can with what you have” rather than, “do it this way or no way” would be more appropriate for Guatemalan healthcare settings where levels of available medical and financial resources vary substantially. [41] Another limitation is that other healthcare professionals (e.g. gynecologists and nurses) that were not part of our sample might also recommend preventive services. Finally, the high recommendation rates seen in all preventive services in Guatemala might be subject to induced-response bias. To reduce this bias, the survey was pilot tested, questions were short, phrased in conditional tense, and reassuring sentences were included. [42].

In conclusion, Guatemalan internal medicine physicians may not adequately recommend preventive services or prioritize them based on cost-effectiveness. These data should be useful in strengthening preventive medicine training. For this LMIC, appropriate use of preventive services can bridge the gap between the increasing NCD incidence and low access to medical treatment.

### Study Registration

clinicaltrials.gov Identifier NCT01515111.

## Supporting Information

Survey Tool S1 (DOC)
