# Supplementary material for: Clinical Preventive Services in Guatemala: A Cross-Sectional Survey of Internal Medicine Physicians
Source: PLoS One. 2012 Oct 31;7(10):e48640. doi: 10.1371/journal.pone.0048640 (PMC3485332; doi:10.1371/journal.pone.0048640)
Supplement: Survey Tool S1 — (DOC) [file pone.0048640.s001.doc]

## Conocimiento y recomendaciones sobre medidas preventivas en el personal de medicina interna de los hospitales escuela de Guatemala

Technical Appendix - Survey Tool.

| Fecha:  _d_ /_m_ /_a_ | Hospital: | # | # de Observador: |
| --- | --- | --- | --- |

| 1. Edad ______ años | | 2. Género ⬜ Masculino  ⬜ Femenino | |
| --- | --- | --- | --- |
| 3. ¿En dónde estudió (o estudia) la carrera de medicina general?          ⬜ USAC ⬜  UFM          ⬜ UMG              ⬜ URL  ⬜ Otra,  Cuál ______________________ , En qué país______________________ | | | |
| 4. Actualmente es: Interno ⬜ | Residente ⬜ | | Médico Internista ⬜ |
| *Marque sólo una* | Si:      ⬜ R1      ⬜ R2      ⬜ R3      ⬜ R4 | | Si:  ⬜ Jefe de Departamento  ⬜ Jefe de Servicio / Unidad  ⬜ Médico-Catedrático  Años de laborar  en este Hospital:  _________ |
| 5. En los últimos 6 meses ¿qué porcentaje de su trabajo dedica a atender pacientes NO HOSPITALIZADOS? *Marque sólo uno*    0 10 20 30 40 50 60 70 80 90 100 | | | |
| 6. En su práctica ¿qué porcentaje de pacientes consultan ASINTOMÁTICOS para un CHEQUEO GENERAL? *Marque sólo uno*  0 10 20 30 40 50 60 70 80 90 100 | | | |

| 7. Enumere de 1 a 6 de acuerdo al número de muertes que causan en Guatemala. *#1 es la que causa más muertes en Guatemala y #6 la que menos causa.*    Enfermedades genéticas ⬜  Enfermedades infecciosas ⬜  Enfermedades perinatales ⬜  Enfermedades cardiovasculares ⬜  Cáncer ⬜  Causas externas (trauma) ⬜ |
| --- |
| 8. Hablando de reducir el impacto de enfermedades crónicas en Guatemala ¿qué tan grande es el efecto de cada una de estas medidas?  Muy Alto Alto Medio Bajo Muy Bajo |
| Avances en el diagnóstico de enfermedades 5 4 3 2 1  Avances en los tratamientos a las enfermedades 5 4 3 2 1  Educación a la población 5 4 3 2 1  Acceso a Servicios Básicos 5 4 3 2 1  (Agua potable, drenajes, etc.)  Cambios en legislaciones 5 4 3 2 1  (Yodar la sal, prohibir fumar en lugares públicos, etc.) |
| 9. ¿Ha escuchado alguna campaña para prevenir enfermedades crónicas en el último año?    ⬜ Si ⬜ No Si sí, ¿Cual?____________________________________ |
| 10. ¿Ha escuchado del Convenio Marco para el Control de Tabaco de la OMS?    ⬜ Si ⬜ No |

| 11. Aproximadamente ¿cuánto tiempo dedica a aconsejar maneras de prevenir enfermedades crónicas por visita?  ____ Nada  ____ 1-2 minutos  ____ 3-5 minutos  ____ 6-10 minutos  ____ 11 o más minutos |
| --- |
| 12. ¿Qué recomendaciones para prevenir enfermedades crónicas conoce? *Puede marcar más de una*  ⬜ Ninguna  ⬜ Guías de las Enfermedades Crónicas No Transmisibles, Ministerio de Salud  ⬜ Normas de Atención en Salud Integral , 1er y 2do Nivel, Ministerio de Salud  ⬜ Instituto Nacional de Cancerología (INCAN)  ⬜ Organización Mundial de la Salud (OMS) para prevenir el cáncer  ⬜ Fuerzas de Servicios Preventivos de Estados Unidos (USPSTF *U S Preventive Services Task Force*)  ⬜ Asociación Médica Americana (AMA)  ⬜ Sociedad Americana de Cáncer (ACS)  ⬜ Asociación Americana del Corazón (AHA)  ⬜ Colegio Americano de Radiología (ACR)  ⬜ Otra, ¿Cuál?  ______________________________________________________________ |

*La siguiente sección es sobre sus recomendaciones para pacientes asintomáticos*

**Fumar**

13. A un paciente ASINTOMÁTICO y SIN FACTORES DE RIESGO, ¿debería preguntarle si fuma?

⬜ Si ⭣ ⬜ No ⭢ *pase a la pregunta 17*

*14. Asumiendo que este paciente viene a chequeo cada año, ¿qué tan frecuentemente debería preguntarlo?*

| Cada año ⬜ | Cada 2 años ⬜ | Cada 3 años ⬜ | Cada 5 años ⬜ | Cada 10 años ⬜ | Una vez en la vida ⬜ |
| --- | --- | --- | --- | --- | --- |

15. Si el paciente fuma, ¿debería ofrecerle consejería para dejarlo?

⬜ Si ⬜ No

16. ¿Qué tratamiento de cesación es el que más le aconsejaría a este paciente?

⬜ Cambio de hábitos (sin tratamiento médico)

⬜  Parches de nicotina

⬜ Chicles de nicotina

⬜ Bupropion (Wellbutrin)

⬜ Vareniclina (Champix)

⬜ Otro_____________________

17. ¿Qué grado de beneficio cree que tiene preguntar si un paciente fuma?

| Beneficio substancial, debe hacerse | Beneficio moderado, debe hacerse | El beneficio es muy pequeño para recomendarlo | No hay beneficio, debemos disuadir su uso | Se ignora el grado de beneficio |
| --- | --- | --- | --- | --- |
| ⬜ | ⬜ | ⬜ | ⬜ | ⬜ |

18. ¿Cuál es la probabilidad que a un paciente le pregunten si fuma en este hospital?

| Muy probable ⬜ | Probable ⬜ | Medio ⬜ | Improbable ⬜ | Casi imposible ⬜ |
| --- | --- | --- | --- | --- |

19. ¿Cuál es la probabilidad que un paciente reciba consejería para dejar de fumar en este hospital?

| Muy probable ⬜ | Probable ⬜ | Medio ⬜ | Improbable ⬜ | Casi imposible ⬜ |
| --- | --- | --- | --- | --- |

20. ¿Cuál es la probabilidad que un paciente reciba medicamentos para dejar de fumar en este hospital?

| Muy probable ⬜ | Probable ⬜ | Medio ⬜ | Improbable ⬜ | Casi imposible ⬜ |
| --- | --- | --- | --- | --- |

**Hábitos Alimenticios**

21. A un paciente ASINTOMÁTICO y SIN FACTORES DE RIESGO, ¿debería darle consejería de conductas alimentarias saludables?

⬜ Si ⭣ ⬜ No ⭢ *pase a la pregunta 23*

*22. Asumiendo que este paciente viene a chequeo cada año, ¿qué tan frecuentemente debería hacerlo?*

| Cada año ⬜ | Cada 2 años ⬜ | Cada 3 años ⬜ | Cada 5 años ⬜ | Cada 10 años ⬜ | Una vez en la vida ⬜ |
| --- | --- | --- | --- | --- | --- |

23. ¿Qué grado de beneficio cree que tiene dar consejería de conductas alimentarias saludables?

| Beneficio sustancial, debe hacerse | Beneficio moderado, debe hacerse | El beneficio es muy pequeño para recomendarlo | No hay beneficio, debemos disuadir su uso | Se ignora el grado de beneficio |
| --- | --- | --- | --- | --- |
| ⬜ | ⬜ | ⬜ | ⬜ | ⬜ |

24. ¿Cuál es la probabilidad que un paciente reciba consejería de conductas alimentarias saludables en este hospital?

| Muy probable ⬜ | Probable ⬜ | Medio ⬜ | Improbable ⬜ | Casi imposible ⬜ |
| --- | --- | --- | --- | --- |

**Perfil Lipídico**

25. A un HOMBRE ASINTOMÁTICO y SIN FACTORES DE RIESGO, ¿debería hacerle un perfil de lípidos?

⬜ Si ⭣ ⬜ No ⭢ *pase a la pregunta 28*

26. A partir de qué edad: _______ años

*27. Asumiendo que este paciente viene a chequeo cada año, ¿qué tan frecuentemente debería hacerlo?*

| Cada año ⬜ | Cada 2 años ⬜ | Cada 3 años ⬜ | Cada 5 años ⬜ | Cada 10 años ⬜ | Una vez en la vida ⬜ |
| --- | --- | --- | --- | --- | --- |

28. ¿Qué grado de beneficio cree que tiene hacer un perfil de lípidos en varones asintomáticos?

| Beneficio sustancial, debe hacerse | Beneficio moderado, debe hacerse | El beneficio es muy pequeño para recomendarlo | No hay beneficio, debemos disuadir su uso | Se ignora el grado de beneficio |
| --- | --- | --- | --- | --- |
| ⬜ | ⬜ | ⬜ | ⬜ | ⬜ |

29. ¿Cuál es la probabilidad que a un paciente asintomático le realicen un perfil de lípidos en este hospital?

| Muy probable ⬜ | Probable ⬜ | Medio ⬜ | Improbable ⬜ | Casi imposible ⬜ |
| --- | --- | --- | --- | --- |

**Presión Arterial**

30. A un HOMBRE ASINTOMÁTICO y SIN FACTORES DE RIESGO, ¿debería medírsele la presión arterial?

⬜ Si ⭣ ⬜ No ⭢ *pase a la pregunta 33*

31. A partir de qué edad: _______ años

*32. Asumiendo que su paciente viene a chequeo cada año, ¿qué tan frecuentemente debería hacerlo?*

| Cada año ⬜ | Cada 2 años ⬜ | Cada 3 años ⬜ | Cada 5 años ⬜ | Cada 10 años ⬜ | Una vez en la vida ⬜ |
| --- | --- | --- | --- | --- | --- |

33. ¿Qué grado de beneficio cree que tiene medir regularmente la presión arterial?

| Beneficio sustancial, debe hacerse | Beneficio moderado, debe hacerse | El beneficio es muy pequeño para recomendarlo | No hay beneficio, debemos disuadir su uso | Se ignora el grado de beneficio |
| --- | --- | --- | --- | --- |
| ⬜ | ⬜ | ⬜ | ⬜ | ⬜ |

34. ¿Cuál es la probabilidad que a un paciente asintomático le midan la presión arterial en este hospital?

| Muy probable ⬜ | Probable ⬜ | Medio ⬜ | Improbable ⬜ | Casi imposible ⬜ |
| --- | --- | --- | --- | --- |

**Riesgo Coronario**

35. A un paciente ASINTOMÁTICO y SIN FACTORES DE RIESGO, ¿debería realizarle un electrocardiograma como tamizaje?

⬜ Si ⭣ ⬜ No ⭢ *pase a la pregunta 37*

*36. Asumiendo que este paciente viene a chequeo cada año, ¿qué tan frecuentemente debería hacerlo?*

| Cada año ⬜ | Cada 2 años ⬜ | Cada 3 años ⬜ | Cada 5 años ⬜ | Cada 10 años ⬜ | Una vez en la vida ⬜ |
| --- | --- | --- | --- | --- | --- |

37. ¿Qué grado de beneficio cree que tiene realizar un electrocardiograma para tamizaje de enfermedad coronaria?

| Beneficio sustancial, debe hacerse | Beneficio moderado, debe hacerse | El beneficio es muy pequeño para recomendarlo | No hay beneficio, debemos disuadir su uso | Se ignora el grado de beneficio |
| --- | --- | --- | --- | --- |
| ⬜ | ⬜ | ⬜ | ⬜ | ⬜ |

38. ¿Cuál es la probabilidad que a un paciente asintomático le realicen un electrocardiograma en este hospital?

| Muy probable ⬜ | Probable ⬜ | Medio ⬜ | Improbable ⬜ | Casi imposible ⬜ |
| --- | --- | --- | --- | --- |

**Tamizaje de Diabetes**

39. A un paciente ASINTOMÁTICO y SIN FACTORES DE RIESGO, ¿debería hacerle tamizaje para diabetes?

⬜ Si ⭣ ⬜ No ⭢ *pase a la pregunta 42*

40. Si marcó sí, ¿Cómo? *Marque sólo la que preferiría*

⬜ Glucosa en ayunas ⬜ Prueba de tolerancia a la glucosa ⬜ Hemoglobina glicosilada

*41. Asumiendo que este paciente viene a chequeo cada año, ¿qué tan frecuentemente debería hacerlo?*

| Cada año ⬜ | Cada 2 años ⬜ | Cada 3 años ⬜ | Cada 5 años ⬜ | Cada 10 años ⬜ | Una vez en la vida ⬜ |
| --- | --- | --- | --- | --- | --- |

42. ¿Qué grado de beneficio cree que tiene hacer tamizaje para diabetes en pacientes asintomáticos sin factores de riesgo?

| Beneficio sustancial, debe hacerse | Beneficio moderado, debe hacerse | El beneficio es muy pequeño para recomendarlo | No hay beneficio, debemos disuadir su uso | Se ignora el grado de beneficio |
| --- | --- | --- | --- | --- |
| ⬜ | ⬜ | ⬜ | ⬜ | ⬜ |

43. ¿Cuál es la probabilidad que a un paciente asintomático le realicen estudios de glucosa o hemoglobina glicosilada en este hospital?

| Muy probable ⬜ | Probable ⬜ | Medio ⬜ | Improbable ⬜ | Casi imposible ⬜ |
| --- | --- | --- | --- | --- |

**Tamizaje de Cáncer Colorrectal**

44. A un paciente ASINTOMÁTICO y SIN FACTORES DE RIESGO, ¿debería hacerle tamizaje para cáncer colorrectal?

⬜ Si ⭣ ⬜ No ⭢ *pase a la pregunta 48*

45. Si marcó sí ¿Cómo? *Marque sólo la que preferiría*

⬜ Sigmoidoscopía ⬜ Colonoscopía ⬜ Sangre oculta en heces (Guayaco)

46. A partir de qué edad: _______ años

*47. Asumiendo que este paciente viene a chequeo cada año, ¿qué tan frecuentemente debería hacerlo?*

| Cada año ⬜ | Cada 2 años ⬜ | Cada 3 años ⬜ | Cada 5 años ⬜ | Cada 10 años ⬜ | Una vez en la vida ⬜ |
| --- | --- | --- | --- | --- | --- |

48. ¿Qué grado de beneficio cree que tiene hacer tamizaje para cáncer colorrectal?

| Beneficio sustancial, debe hacerse | Beneficio moderado, debe hacerse | El beneficio es muy pequeño para recomendarlo | No hay beneficio, debemos disuadir su uso | Se ignora el grado de beneficio |
| --- | --- | --- | --- | --- |
| ⬜ | ⬜ | ⬜ | ⬜ | ⬜ |

49. ¿Cuál es la probabilidad que a un paciente le realicen estudios para prevenir cáncer colorrectal en este hospital?

| Muy probable ⬜ | Probable ⬜ | Medio ⬜ | Improbable ⬜ | Casi imposible ⬜ |
| --- | --- | --- | --- | --- |

**Tamizaje de Cáncer de Próstata**

50. A un paciente ASINTOMÁTICO y SIN FACTORES DE RIESGO, ¿debería hacerle tamizaje para cáncer de próstata?

⬜ Si ⭣ ⬜ No ⭢ *pase a la pregunta 54*

51. Si marcó sí ¿Cómo? *Marque sólo la que preferiría*

⬜ Antígeno prostático ⬜ Examen rectal digital

52. A partir de qué edad: _______ años

*53. Asumiendo que este paciente viene a chequeo cada año, ¿qué tan frecuentemente debería hacerlo?*

| Cada año ⬜ | Cada 2 años ⬜ | Cada 3 años ⬜ | Cada 5 años ⬜ | Cada 10 años ⬜ | Una vez en la vida ⬜ |
| --- | --- | --- | --- | --- | --- |

54. ¿Qué grado de beneficio cree que tiene hacer tamizaje para cáncer de próstata?

| Beneficio sustancial, debe hacerse | Beneficio moderado, debe hacerse | El beneficio es muy pequeño para recomendarlo | No hay beneficio, debemos disuadir su uso | Se ignora el grado de beneficio |
| --- | --- | --- | --- | --- |
| ⬜ | ⬜ | ⬜ | ⬜ | ⬜ |

55. ¿Cuál es la probabilidad que a un paciente le realicen estudios para prevenir cáncer de próstata en este hospital?

| Muy probable ⬜ | Probable ⬜ | Medio ⬜ | Improbable ⬜ | Casi imposible ⬜ |
| --- | --- | --- | --- | --- |

**Tamizaje de Cáncer de Cérvix**

56. A una mujer ASINTOMÁTICA y SIN FACTORES DE RIESGO, ¿debería hacerle tamizaje para cáncer de cérvix?

⬜ Si ⭣ ⬜ No ⭢ *pase a la pregunta 60*

57. Si sí ¿Cómo? *Marque sólo la que preferiría*

⬜ Papanicolau ⬜ Inspección directa con ácido acético

58. A partir de qué edad: _______ años

*59. Asumiendo que esta paciente viene a chequeo cada año, ¿qué tan frecuentemente debería hacerlo?*

| Cada año ⬜ | Cada 2 años ⬜ | Cada 3 años ⬜ | Cada 5 años ⬜ | Cada 10 años ⬜ | Una vez en la vida ⬜ |
| --- | --- | --- | --- | --- | --- |

60. ¿Qué grado de beneficio cree que tiene hacer tamizaje para cáncer de cérvix?

| Beneficio sustancial, debe hacerse | Beneficio moderado, debe hacerse | El beneficio es muy pequeño para recomendarlo | No hay beneficio, debemos disuadir su uso | Se ignora el grado de beneficio |
| --- | --- | --- | --- | --- |
| ⬜ | ⬜ | ⬜ | ⬜ | ⬜ |

61. ¿Cuál es la probabilidad que a una paciente le realicen estudios para prevenir cáncer de cérvix en este hospital?

| Muy probable ⬜ | Probable ⬜ | Medio ⬜ | Improbable ⬜ | Casi imposible ⬜ |
| --- | --- | --- | --- | --- |

**Tamizaje de Cáncer de Mama**

62. A una mujer ASINTOMÁTICA y SIN FACTORES DE RIESGO, ¿debería hacerle tamizaje para cáncer de mama?

⬜ Si ⭣ ⬜ No ⭢ *pase a la pregunta 66*

63. Si sí ¿Cómo? *Marque sólo la que preferiría*

⬜ Mamografía ⬜ Examen clínico de mamas

64. A partir de qué edad: _______ años

*65. Asumiendo que esta paciente viene a chequeo cada año, ¿qué tan frecuentemente debería hacerlo?*

| Cada año ⬜ | Cada 2 años ⬜ | Cada 3 años ⬜ | Cada 5 años ⬜ | Cada 10 años ⬜ | Una vez en la vida ⬜ |
| --- | --- | --- | --- | --- | --- |

66. ¿Qué grado de beneficio cree que tiene hacer tamizaje para cáncer de mama?

| Beneficio sustancial, debe hacerse | Beneficio moderado, debe hacerse | El beneficio es muy pequeño para recomendarlo | No hay beneficio, debemos disuadir su uso | Se ignora el grado de beneficio |
| --- | --- | --- | --- | --- |
| ⬜ | ⬜ | ⬜ | ⬜ | ⬜ |

67. ¿Cuál es la probabilidad que a una paciente le realicen estudios para prevenir cáncer de mama en este hospital?

| Muy probable ⬜ | Probable ⬜ | Medio ⬜ | Improbable ⬜ | Casi imposible ⬜ |
| --- | --- | --- | --- | --- |

| 68. EN GENERAL usted considera que sus recomendaciones son  ⬜ más frecuentes ⬜ similares ⬜ menos frecuentes  que las guías de prevención que utiliza  *Hay muchas razones por las que los médicos no recomiendan estas medidas preventivas.*  69. De la lista siguiente, diga ¿qué tan frecuentemente estas razones le impiden recomendar dichas medidas preventivas?   |  | Siempre | Casi siempre | A veces | Casi nunca | Nunca | | --- | --- | --- | --- | --- | --- | | No tengo suficiente tiempo | 1 | 2 | 3 | 4 | 5 | | No me siento entrenado para dar estas recomendaciones | 1 | 2 | 3 | 4 | 5 | | A los pacientes no les interesa | 1 | 2 | 3 | 4 | 5 | | Estas medidas no son muy útiles | 1 | 2 | 3 | 4 | 5 | | Se me olvida | 1 | 2 | 3 | 4 | 5 | | Creo que los pacientes no los van a poder pagar | 1 | 2 | 3 | 4 | 5 | | |
| --- | --- | --- | --- | --- | --- | --- | --- | --- | --- | --- | --- | --- | --- | --- | --- | --- | --- | --- | --- | --- | --- | --- | --- | --- | --- | --- | --- | --- | --- | --- | --- | --- | --- | --- | --- | --- | --- | --- | --- | --- | --- | --- | --- |
| 70. ¿Quién debería ser responsable de implementar un programa nacional para prevenir enfermedades crónicas?  *Marque sólo uno* | |
| ⬜ Ministerio de Salud  ⬜ Hospitales Públicos  ⬜ IGSS  ⬜ Universidades | ⬜ Personal Médico de los Hospitales  ⬜ Colegio de Médicos de Guatemala  ⬜ Organizaciones No Gubernamentales ONGs  ⬜ Otros, mencione  _____________________________________ |
| 71. ¿Quién debería tomar la iniciativa para mejorar la educación en medicina preventiva de los médicos de Guatemala?  *Marque sólo uno*. | |
| ⬜ Ministerio de Salud  ⬜ Hospitales Públicos  ⬜ IGSS  ⬜ Universidades | ⬜ Personal Médico de los Hospitales  ⬜ Colegio de Médicos de Guatemala  ⬜ Organizaciones No Gubernamentales ONGs  ⬜ Otros, mencione  _____________________________________ |

*Usted…*

| 72. ¿Ha probado o experimentado fumar, aunque sea un sólo jalón?  ⬜ Sí ⬜ No  73. ¿Ha fumado al menos 100 cigarros durante su vida?  ⬜ Si ⭣ ⬜ No ⭢ *pase a la pregunta 78*    74. ¿Cuántos años tenía cuando comenzó a fumar?    _________ años ⬜ No sé / No estoy seguro    75. En los últimos 30 días, usted ha fumado…    ⬜ Todos los días ⬜ Algunos días ⬜ No he fumado en los últimos 30 días  76. Si ha dejado de fumar ¿cuánto tiempo hace desde que dejó de hacerlo?    _________ años ⬜ No sé / No estoy seguro  77. ¿Cuántos cigarros por día solía/suele fumar?  _________ por día. ⬜ No sé / No estoy seguro. |
| --- |
| 78. Vive con alguien que tenga o sea: *Puede marcar más de una*            ⬜ Fumador ⬜ Alcohólico  ⬜ Cáncer ⬜ Cualquier enfermedad cardiovascular      ⬜ Diabetes              ⬜ Insuficiencia Renal Crónica  ⬜ Enfermedad Pulmonar Crónica  ⬜ Otra Enfermedad Crónica  Especifique ____________________________________  ⬜ No |

**Gracias por completar esta encuesta. Apreciamos sinceramente su participación en este estudio.**
